# An Experimental Research into Surgical Shock

**Published:** 1899-07

**Authors:** 


An Experimental Research into Surgical Shock. An Essay Awarded the Cartwright Prize for 1897. By George W. Crile, A. M., M. D., Ph. D., Professor of the Principles of Surgery and Applied Anatomy in the Cleveland College of Physicians and Surgeons; formerly Professor of Physiology in the Medical Department of the University of Wooster; Attending Surgeon to the St. Alexis and Cleveland General Hospitals. Philadelphia: J. B. Lippincott Company. 1899.

This work, which was awarded the Cartwright prize for 1897, is based upon the experiments conducted upon 148 unselected animals (dogs). From the complete records of each experiment sufficient have been taken to illustrate the text. The author, in his modest introduction, does not assume to have touched upon all the phases of the subject, nor to have exhausted the possibilities of those receiving the most careful attention. Nevertheless, he has produced an admirable work full of data and suggestiveness, which will prove useful to the surgeon and the physiologist.

Beginning with a historical account of the theories concerning shock, the writer follows with a description of the modes of investigation and annotations employed in the conduct of the experiments, which follow next, and form the bulk of the work. The author then considers the effect of injury to the various tissues and organs of the body upon the production of shock and other factors, such as duration of operation, temperature, physical condition, anesthesia, hemorrhage, vaso-motor changes and cardiac changes. The post mortem appearances are next dwelt upon, followed by advice as to the prevention and treatment of shock.

Among the beneficial agents in combating shock the author enumerates strychnia sulphate, artificial respiration, intravenous infusion of normal saline solution, application of heat, and a dependent posture of the head. Among his valuable conclusions the author states his belief that surgical shock is mainly due to impairment or breakdown of the vaso-motor mechanism, and gives cogent reasons for his position in the matter. The work throughout bears evidence of great care and accuracy in both the conduct of the experiments and the deductions to be drawn therefrom. The book is admirably printed and the illustrations are very clear.

